# Revision of the world species of the genus *Habroteleia* Kieffer (Hymenoptera, Platygastridae, Scelioninae)

**DOI:** 10.3897/zookeys.730.21846

**Published:** 2018-01-17

**Authors:** Hua-yan Chen, Elijah J. Talamas, Lubomír Masner, Norman F. Johnson

**Affiliations:** 1 Department of Entomology, The Ohio State University, 1315 Kinnear Road, Columbus, Ohio 43212, U.S.A.; 2 Florida Department of Agriculture and Consumer Services, The Doyle Conner Building, 1911 SW 34th St, Gainesville, Florida 32608, U.S.A.; 3 Agriculture and Agri-Food Canada, K.W. Neatby Building, Ottawa, Ontario K1A 0C6, Canada; 4 Department of Evolution, Ecology and Organismal Biology, The Ohio State University, 1315 Kinnear Road, Columbus, Ohio 43212, U.S.A.

**Keywords:** Platygastroidea, identification key, species description

## Abstract

The genus *Habroteleia* Kieffer is revised. Seven species are recognized, three are redescribed: *H. flavipes* Kieffer, *H. persimilis* (Kozlov & Kononova), *H. ruficoxa* (Kieffer); and four species are described as new: *H. mutabilis* Chen & Talamas, **sp. n.**, *H. salebra* Chen & Talamas, **sp. n.**, *H. soa* Chen & Talamas, **sp. n.**, and *H. spinosa* Chen & Johnson, **sp. n.** Four species are treated as junior synonyms of *Habroteleia flavipes* Kieffer: *Chrestoteleia bakeri* Kieffer, **syn. n.**, *Habroteleia bharatensis* Saraswat, **syn. n.**, *Habroteleia browni* Crawford, **syn. n.**, and *Habroteleia kotturensis* (Sharma), **syn. n.** *Habroteleia dagavia* (Kozlov & Lê), **syn. n.** is treated as junior synonym of *Habroteleia persimilis* (Kozlov & Kononova). *Baryconus vindhiensis* (Sharma), **comb. n.** is transferred out of *Habroteleia* Kieffer. *Habroteleia impressa* (Kieffer) and *H. scapularis* (Kieffer) remain valid species but their identity and status are unclear.

## Introduction

The genus *Habroteleia* was originally described by Kieffer (1905) based on the type species, *Habroteleia flavipes* Kieffer, collected on the island of Sumatra, Indonesia. Kieffer (1913) later proposed *Chrestoteleia* for a single species, *Chrestoteleia bakeri* Kieffer, collected from the Philippines, which was treated by Baltazar (1961) as a junior synonym of *Habroteleia*. Nine species have since been described from India, Japan and the Philippines. We here provide the first comprehensive treatment of the genus, including examination of type specimens of all species except *H. impressa* (Kieffer) and *H. scapularis* (Kieffer), for which we were unable to locate type material. The previously described species of *Habroteleia* were recorded from the Oriental region, extending from India to Japan, and we here provide records that expand the distribution of *Habroteleia* to include Madagascar, Papua New Guinea, and the Fijian archipelago.

The host of *Habroteleia* is unknown, but we suspect that it parasitizes orthopteran eggs (large and elongate) based on its elongate body and because Orthoptera is suspected to be the plesiomorphic host group for the platygastroids as a whole (Austin et al. 2005).

The contributions of the authors are as follows. H.-Y. Chen, E. J. Talamas and N.F. Johnson: character definition, generic concept development, species concept development, imaging, key development, manuscript preparation; L. Masner: character definition, generic concept development, species concept development. The authors of the new species are indicated in the heading of each description.

## Materials and methods

This work is based upon specimens in the following collections, with abbreviations used in the text: BPBM, Bernice P. Bishop Museum, Honolulu, HI; CNCI, Canadian National Collection of Insects, Ottawa, Canada; CAS, California Academy of Sciences, San Francisco, CA; FSCA, Florida State Collection of Arthropods, Gainesville, FL; IEBR, Institute of Ecology and Biological Resourves, Hanoi, Vietnam; MCSN, Museo Civico de Storia Naturale “Giacomo Doria”, Genoa, Italy; MNHN, Muséum National d’Histoire Naturelle, Paris, France; OSUC, C.A. Triplehorn Insect Collection, Ohio State University, Columbus, OH; SCAU, South China Agricultural University, Guangzhou, China; UCDC, R.M. Bohart Museum of Entomology, University of California, Davis, CA; ZIN, Zoological Museum, Academy of Sciences, St. Petersburg, Russia.

Abbreviations and morphological terms used in text: A1, A2, ... A12: antennomere 1, 2, … 12; claval formula: distribution of the large, multiporous basiconic sensilla on the underside of apical antennomeres of the female, with the segment interval specified followed by the number of sensilla per segment (Bin, 1981); EH: eye height, length of compound eye measured parallel to dorsoventral midline of head; IOS: interocular space, minimal distance on frons between compound eyes; OD: ocellar diameter, greatest width of ocellus; OOL: ocular ocellar line, shortest distance from inner orbit and outer margin of posterior ocellus (Masner 1980); T1, T2, ... T7: metasomal tergite 1, 2, ... 7; S1, S2, … S7: metasomal sternite 1, 2, … 7. Morphological terminology otherwise generally follows Masner (1980) and Mikó et al. (2007).

Morphological terms used in this work were as in the Hymenoptera Anatomy Ontology (Yoder et al. 2010) (Appendix 1). Identifiers (URIs) in the format HAO_XXXXXXX represent concepts in the HAO and are provided to enable readers to confirm their understanding of the concepts being referenced. To learn more about a given concept, including additional images, notes, references and other metadata, use the identifier as a search term at http://glossary.hymao.org or use the identifier as a web-link.

In the Material Examined section the metadata for the specimens studied are recorded in an abbreviated format, using unique identifiers (numbers prefixed with “OSUC”, “CASENT”, “FBA”, “MNHN_EY”) for the individual specimens. The label data for all specimens have been georeferenced and recorded in the Hymenoptera Online database, and details on the data associated with these specimens can be accessed at the following link, hol.osu.edu, and entering the identifier in the form (note the space between the acronym and the number). The electronic version of the paper contains hyperlinks to external resources. Insofar as possible, the external information conforms to standards developed and maintained through the organization Biodiversity Information Standards (Taxonomic Database Working Group). All new species have been prospectively registered with Zoobank (Polaszek et al. 2005, www.zoobank.org), and other taxonomic names, where appropriate, have been retrospectively registered. The external hyperlinks are explicitly cited in the endnotes so that users of the printed version of this article have access to the same resources.

Data associated with the genus *Habroteleia* can be accessed at http://hol.osu.edu/index.html?id=488. The generic and species descriptions were generated by a database application, vSysLab (vsyslab.osu.edu), designed to facilitate the production of a taxon by character data matrices, and to integrate those data with the existing taxonomic, media, and specimen-level database. Data may be exported in both text format and as input files for other applications. The text output for descriptions is in the format of “Character: Character state (s)”. Polymorphic characters are indicated by semicolon-separated character states.

Images and measurements were produced with multiple systems. Photographs of IEBR specimens were captured with a Canon Rebel 600 camera connected to a Wild M10 microscope with a Fotoprojektiv 2.5×/SLR 10446175 adapter and stacked with the program Zerene Stacker. A scale bar was calibrated for images taken at the maximum magnification of the microscope. The remaining images were produced with Combine ZP and AutoMontage extended-focus software, using a JVC KY-F75U digital camera, Leica Z16 APOA microscope, and 1X objective lens. Images were post-processed with Abobe Photoshop CS3 Extended. A standard set of images is provided for each species: dorsal habitus, lateral habitus, dorsal and lateral views of the head and mesosoma, and anterior view of head. The individual images are archived in Specimage (specimage.osu.edu), the image database at The Ohio State University.

Images of primary types of *H. ruficoxa* and *H. persimilis* were provided by Agnièle Touret-Alby (MNHN) and Konstantin Samartsev (ZIN), respectively. Images of the primary type of *Baryconus vindhiensis*, *Habroteleia bharatensis* and *Habroteleia kotturensis* were made available by Talamas et al. (2017) and images of *Triteleia dagavia* were made available by Talamas and Pham (2017), all are used in this publication with permission.

## Taxonomy

### Habroteleia

Taxon classificationAnimaliaHymenopteraPlatygastridae

Kieffer

Habroteleia Kieffer, 1905: 14 (original description. Type: Habroteleia flavipes Kieffer, by monotypy); Kieffer 1908: 114 (keyed); Brues 1908: 27, 38 (diagnosis, list of species, keyed); Kieffer 1910: 63, 69 (description, list of species, keyed); Kieffer 1913: 220 (description); Kieffer 1926: 267, 363 (description, keyed, key to species); Muesebeck and Walkley 1956: 357 (citation of type species); Baltazar 1961: 395 (synonymy); Baltazar 1966: 177 (cataloged, catalog of species of the Philippines); Masner 1976: 10, 26 (description, keyed); Mani and Sharma 1982: 155, 167 (description, keyed); Johnson 1992: 398 (cataloged, catalog of world species); Austin and Field 1997: 24, 68 (structure of ovipositor system, discussion of phylogenetic relationships, genus misplaced in Calliscelionini); Lê 2000: 31 (keyed); Kononova and Kozlov 2008: 23, 255 (description, keyed); Chen et al. 2013: 11 (keyed).
http://zoobank.org/CBFA7C74-68DD-44F2-BE05-AEBD88E6FA8D
http://bioguid.osu.edu/xbiod_concepts/488
Chrestoteleia Kieffer, 1913: 388 (original description. Type: Chrestoteleia bakeri Kieffer, by monotypy and original designation. Synonymized by Baltazar (1961)); Kieffer 1926: 271, 442 (description, keyed, key to species); Muesebeck and Walkley 1956: 342 (citation of type species); Baltazar 1961: 395 (junior synonym of Habroteleia Kieffer); Baltazar 1966: 182 (cataloged, catalog of species of the Philippines).
http://zoobank.org/4EA90A05-D50A-42BF-B1C0-852F4B56FCBA
http://bioguid.osu.edu/xbiod_concepts/8933
Crestoteleia Kieffer: Kieffer 1916: 180 (key to new species described from the Philippines, spelling error).

#### Description.

Length 2.18–5.18 mm; body moderately to markedly elongate, robust.

***Head.*** Head shape in dorsal view: transverse. Hyperoccipital carina: absent. Occipital carina: present, complete or broadly interrupted medially. Anterior margin of occipital carina: crenulate. OOL: lateral ocellus nearly contiguous with inner orbits, OOL < 0.5 OD; lateral ocellus contiguous with inner orbit. Upper frons: convex, without frontal shelf or carina. Antennal scrobe: broadly convex or conave medially with distinct depression. Sculpture of antennal scrobe: smooth to punctate. Submedian carina: absent. Orbital carina: absent. Inner orbits: diverging ventrally. IOS/EH: IOS distinctly less than EH. Interantennal process: short, often excavate medially. Central keel: present or absent. Antennal foramen: oriented laterally on interantennal process. Facial striae: absent. Malar sulcus: present. Setation of compound eye: absent. Gena: broad, convex, distinctly produced behind eye. Clypeus shape: narrow, slightly convex medially, lateral corners not produced. Anterior (or ventral) margin of clypeus: straight. Anteclypeus: absent. Postclypeus: absent. Labrum: not visible in anterior view. Number of mandibular teeth: 2. Arrangement of mandibular teeth: transverse. Number of maxillary palpomeres: 4. Shape of maxillary palpomeres: cylindrical. Number of labial palpomeres: 2.

***Antenna.*** Number of antennomeres in female: 12. Number of antennomeres in male: 12. Insertion of radicle into A1: parallel to longitudinal axis of A1. Shape of A1: more or less cylindrical, not flattened. Length of A3 of female: distinctly longer than A2. Number of clavomeres in female antenna: 6. Number of antennomeres with multiporous plate sensilla in female: 5. Arrangement of doubled multiporous plate sensilla on female clava: in longitudinal pairs. Number of antennomeres bearing tyloids in male antenna: 0. Shape of male flagellum: filiform.

***Mesosoma.*** Transverse pronotal carina: present anterior to epomial carina, present or absent posterior to epomial carina. Posterior apex of pronotum in dorsal view: straight, bifid apically to articulate with tegula. Epomial carina: present. Anterior face of pronotum: oblique, visible dorsally, short. Lateral face of pronotum: weakly concave below position of dorsal epomial carina. Netrion: present. Netrion shape: moderately wide, open ventrally. Anterior portion of mesoscutum: vertical, flexed ventrally to meet pronotum. Mesoscutum shape: pentagonal, excavate at base of wings. Skaphion: absent. Notauli: present, percurrent. Parapsidal lines: absent. Antero-admedian lines: absent. Transscutal articulation: well-developed, narrow. Shape of mesoscutellum: trapezoidal. Lateral mesoscutellar spine: absent. Median mesoscutellar spine: absent. Axillular spine: absent. Surface of mesoscutellum: convex throughout. Median longitudinal furrow on mesoscutellum: absent; present. Metascutellum: clearly differentiated. Form of metascutellum: transverse. Posterior margin of metascutellum: straight with a small projection medially. Setation of metascutellum: absent. Metapostnotum: not defined externally. Lateral propodeal projection: present. Median propodeal projection: present. Mesopleural carina: present. Mesal course of acetabular carina: not separating fore coxae. Mesopleural pit: present. Posterodorsal corner of mesopleuron: rounded anteriorly.

***Legs.*** Number of mesotibial spurs: 1. Number of metatibial spurs: 1. Dorsal surface of metacoxa: smooth; punctate. Shape of metacoxa: cylindrical, ecarinate. Trochantellus: indicated by transverse sulcus on femur.

***Wings.*** Wing development of female: macropterous. Wing development of male: macropterous. Tubular veins in fore wing: present. Bulla of fore wing R: absent. Length of marginal vein of fore wing: more than twice as long as stigmal vein. Origin of r-rs in fore wing: arising from marginal vein along costal margin. Basal vein (Rs+M) in fore wing: absent. Development of R vein in hind wing: complete.

***Metasoma.*** Number of external metasomal tergites in female: 6. Number of external metasoma sternites in female: 6. Number of external metasomal tergites in male: 8. Number of external metasomal sternites in male: 8. Shape of metasoma: lanceolate. Laterotergites: present, narrow. Laterosternites: present. T1 of female: flat; medially convex as a small hump anteriorly. Relative size of metasomal segments: T3 longest, T2 and T4 subequal in length. Metasomal tergites with basal crenulae: T2. Sublateral carinae on tergites: absent. Median longitudinal carina on metasomal terga: absent. Shape of female T6: flattened. Anterior margin of S1: not produced anteriorly, straight. Felt fields: absent. Ovipositor: *Ceratobaeus*-type (Austin and Field 1997).

#### Diagnosis.

*Habroteleia* can be separated from other scelionines by the combination of the following characters: epomial carina present; malar and facial striae absent; marginal vein many times longer than stigmal vein; postmarginal vein (R1) absent or rudimentary; propodeum with lateral and median projections; T6 in females strongly depressed dorsoventrally to form a flat triangle; male antenna without tyloid (Chen et al. 2013).

The wing venation and large size of *Habroteleia* make it a relatively easy genus to identify. In all species of *Habroteleia* the marginal vein is many times longer than the stigmal vein and the postmarginal vein is very short or absent. *Macroteleia* and *Triteleia* share the presence of a long marginal vein, though in the latter genus it is variable and the marginal and stigmal veins can be of similar length. However, both *Macroteleia* and *Triteleia* have a well-developed postmarginal vein. *Habroteleia* also differs from these genera in that it has a *Ceratobaeus*-type ovipositor (Austin and Field 1997). The complexity of this system suggests that while these three genera are quite similar in external appearance, in fact they may not be closely related at all. Alternatively, it implies that the ovipositor system is much more labile than expected. Unfortunately, *Habroteleia* was not included among the taxa in the phylogenetic analysis of Murphy et al. (2007), and we therefore do not have an independent assessment of its relations. The structure of the ovipositor is of limited use for separating *Habroteleia* from *Triteleia* because it is rarely extruded in preserved specimens of the latter, and it is not obvious from external morphology (e.g. visibility of T7 in females) that *Habroteleia* has a *Ceratobaeus*-type ovipositor. Chen et al. (2013) provided a key to separate these genera which we here present again.

### Key to separate *Macroteleia*, *Triteleia* and *Habroteleia*

**Table:** 

1	Postmarginal vein in fore wing absent or rudimentary; ovipositor *Ceratobaeus*-type	***Habroteleia* Kieffer**
–	Postmarginal vein in fore wing well developed, distinctly longer than stigma vein (r-rs); ovipositor *Scelio*-type	**2**
2	Female T6 strongly compressed laterally, wedge-like; male apical tergite apically emarginate or with 1 central spine but never bispinose	***Macroteleia* Westwood**
–	Female T6 triangular, not compressed laterally; male apical tergite with posterolateral conrners bispinose or at least pointed	***Triteleia* Kieffer**

### Key to females

(unknown for *H. ruficoxa* (Kieffer))

**Table:** 

1	T1 with horn (Figs 14, 20, 26, 32, 38, 62, 76)	**2**
–	T1 without horn (Figs 16, 22, 79, 87)	**4**
2	Posterior vertex largely smooth with sparse to moderate punctures above occipital carina (Fig. 74); gena sparsely punctate (Fig. 72); mesepisternum anteroventral to mesopleural depression largely smooth with sparse punctures (Fig. 72)	***Habroteleia salebra* Chen & Talamas, sp. n.**
–	Posterior vertex densely punctate to punctate rugose (Figs 13, 20, 26, 32, 38, 61); gena densely punctate to punctate rugose (Figs 12, 18, 24, 30, 36, 60); mesepisternum anteroventral to mesopleural depression densely punctate to punctate rugose (Figs 12, 18, 24, 30, 36, 60)	**3**
3	Median propodeal projection short (Figs 14, 20, 26, 32, 38); T6 in female longitudinally striate, with fine punctures in interstices (Fig. 88)	***Habroteleia flavipes* Kieffer**
–	Median propodeal projection long (Figs 56, 62); T6 in female densely punctate and without longitudinal striae (Fig. 89)	***Habroteleia persimilis* (Kozlov & Kononova)**
4	Central keel of frons present (Figs 80, 86); upper frons densely punctate (Figs 80, 86); transverse sulcus on T2 present (Figs 79, 87)	**5**
–	Central keel of frons absent (Figs 45, 51); upper frons sparsely punctate (Figs 45, 51); transverse sulcus on T2 absent (Figs 16, 22)	***Habroteleia mutabilis* Chen & Talamas, sp. n.**
5	Apex of T6 in female rounded (Fig. 81); posterior vertex punctate rugose (Fig. 80)	***Habroteleia soa* Chen & Talamas, sp. n.**
–	Apex of T6 in female with small spine (Fig. 5); posterior vertex smooth with sparse punctures (Fig. 85)	***Habroteleia spinosa* Chen & Johnson, sp. n.**

### Key to males

**Table:** 

1	Apex of T8 with apical spine (Fig. 3)	**2**
–	Apex of T8 without apical spine (Fig. 4)	**4**
2	Occipital carina interrupted medially (Fig. 74); posterior vertex largely smooth with sparse to moderate punctures above occipital carina (Fig. 74); gena sparsely punctate (Fig. 72)	***Habroteleia salebra* Chen & Talamas, sp. n.**
–	Occipital carina complete (Figs 14, 20, 26, 32, 38, 80); posterior vertex densely punctate or punctate rugose (Figs 14, 20, 26, 32, 38, 79); gena densely punctate or punctate rugose (Figs 12, 24, 30, 36, 78)	**3**
3	Central keel absent (Figs 21, 27); netrion rugulose anteriorly, smooth posteriorly, sometimes smooth only along posterior margin (Fig. 6); T1 densely longitudinally striate with rugulose interstices (Figs 22, 34, 40)	***Habroteleia flavipes* Kieffer**
–	Central keel present (Fig. 80); netrion coarsely striate (Fig. 78); T1 sparsely longitudinally striate, smooth in interstices (Fig. 79)	***Habroteleia soa* Chen & Talamas, sp. n.**
4	Central keel absent (Figs 45, 51); transverse sulcus on T2 absent (Figs 46, 52)	***Habroteleia mutabilis* Chen & Talamas, sp. n.**
–	Central keel present (Figs 57, 69, 86); transverse sulcus on T2 present (Figs 64, 70, 87)	**5**
5	Median propodeal projection long (Figs 56, 60, 62); notaulus formed by contiguous punctures (Figs 56, 61)	***Habroteleia persimilis* (Kozlov & Kononova)**
–	Median propodeal projection short (Figs 66, 85); notaulus formed by discrete punctures (Figs 68, 85)	**7**
7	Posterior vertex punctate rugose (Fig. 68); mesoscutal midlobe densely punctate (Fig. 68); gena punctate rugose throughout (Fig. 66)	***Habroteleia ruficoxa* (Kieffer)**
–	Posterior vertex smooth with sparse punctures (Fig. 85); mesoscutal midlobe densely and finely punctate along anterior margin, otherwise smooth (Fig. 85): gena sparsely punctate (Fig. 83)	***Habroteleia ruficoxa* Chen & Johnson, sp. n.**

### Baryconus vindhiensis

Taxon classificationAnimaliaHymenopteraPlatygastridae

(Sharma) comb. n.

http://zoobank.org/0EC31368-F49B-4183-B2C5-BD09C1C07753

http://bioguid.osu.edu/xbiod_concepts/4540

Figures 7–10

Triteleia vindhiensis Sharma, 1981: 451 (original description); Mani and Sharma 1982: 168 (description, generic transfer).
Habroteleia vindhiensis (Sharma): Johnson 1992: 399 (cataloged, type information).

#### Link to distribution map.

[http://hol.osu.edu/map-large.html?id=4540]

#### Material examined.

Holotype, female, *T. vindhiensis*: **INDIA**: Madhya Pradesh St., 21.5, Panna-Satna Road, 9.IX–10.IX.1979, M. S. Mani et al., USNMENT01197073 (deposited in USNM).

#### Comments.

The deep frontal depression margined by a sharp carina (Fig. 8), pronounced occiput (Fig. 9), long postmarginal vein and short marginal vein (Fig. 9) clearly indicate that this species belongs to *Baryconus*.

### Habroteleia flavipes

Taxon classificationAnimaliaHymenopteraPlatygastridae

Kieffer

http://zoobank.org/ACD49F55-9F4E-4C91-A044-22DCC0428FF6

http://bioguid.osu.edu/xbiod_concepts/4535

Figures 6 , 11–16 , 17–22 , 23–28 , 29–34 , 35–40 , 88

Habroteleia flavipes Kieffer, 1905: 15 (original description, keyed); Kieffer 1926: 363 (description, keyed); Bin 1974: 455 (type information); Johnson 1992: 399 (cataloged, type information).
Habroteleia browni Crawford, 1910: 125 (original description); Kieffer 1926: 363, 364 (description, keyed); Baltazar 1966: 177 (cataloged, synonymy, type information, distribution); Masner and Muesebeck 1968: 37 (type information); Johnson 1992: 399 (cataloged, type information), **syn. n.**
http://zoobank.org/EC09DB18-92D9-4FB1-B986-3F7EAD7D54E4
http://bioguid.osu.edu/xbiod_concepts/4534
Chrestoteleia Bakeri Kieffer, 1913: 389 (original description); Kelner-Pillault 1958: 150 (type information); Johnson 1992: 399 (type information), **syn. n.**
http://zoobank.org/F18A3905-9A5A-4755-A56A-5379E8564044
http://bioguid.osu.edu/xbiod_concepts/8935
Chrestoteleia bakeri Kieffer: Kieffer, 1926: 443 (description, keyed); Baltazar 1966: 177 (junior synonym of Habroteleia browni Crawford); Baltazar 1966: 182 (cataloged, type information, distribution).
Habroteleia bakeri (Kieffer): Baltazar 1961: 395 (generic transfer, diagnosis).
Habroteleia bharatensis Saraswat, 1978: 7 (original description); Mani and Sharma 1982: 167 (description); Johnson 1992: 398 (cataloged), **syn. n.**
http://zoobank.org/309A96B1-1DCA-45CA-B1AB-1D6E570C7E07
http://bioguid.osu.edu/xbiod_concepts/4533
Triteleia kotturensis Sharma, 1981: 447 (original description), **syn. n.**
http://zoobank.org/28DFECE9-8723-4ACA-BB61-96B11C9546A8
http://bioguid.osu.edu/xbiod_concepts/8940
Habroteleia kotturensis (Sharma): Mani and Sharma 1982: 168 (description, generic transfer); Johnson 1992: 399 (cataloged, type information)

#### Description.

Body length of female: 4.36–4.72 mm (n=20). Body length of male: 4.15–4.52 mm (n=20). Length of A3 in male: longer than A2. Punctation of frons above antennal scrobe: dense. Sculpture of antennal scrobe: punctate rugose to smooth. Central keel: absent. Sculpture of ventrolateral frons: punctate rugose. Occipital carina: complete. Sculpture of posterior vertex: densely punctate to punctate rugose. Sculpture of gena: densely punctate to punctate rugose. Sculpture of occiput: punctate rugose.

Color of mesosoma: black. Sculpture of dorsal pronotal area: punctate rugose. Sculpture of lateral pronotal area: densely punctate. Sculpture of netrion: anterior half rugulose, posterior half smooth. Setae of netrion: dense throughout. Sculpture of notaulus: contiguously punctate. Sculpture of mesoscutal midlobe: largely punctate rugose, with a medial furrow and smooth areas laterally. Sculpture of lateral lobe of mesoscutum: sparsely punctate. Sculpture of lateral propodeal area: rugose. Setation of mesoscutellum: dense. Sculpture of mesoscutellum: coarsely punctate rugose. Median propodeal projection: short. Mesopleural carina: distinct. Sculpture of mesepisternum anteroventral to mesopleural depression: punctate rugose. Sculpture of dorsal metapleural area: smooth to rugulose. Sculpture of ventral metapleural area: punctate rugose. Setation of ventral metapleural area: dense. Color of legs: orange-yellow to dark brown; dark brown to black. Sculpture of hind coxa: densely punctate.

Color of metasoma: black; black with T3–T4 and S2–S5 partly brown to yellow. T1 horn in female: present. Sculpture of posterior margin of T1 in female: densely longitudinally striate, punctate rugulose in interstices. Transverse sulcus on T2: present. Sculpture of T2–T5: densely longitudinally striate, with fine punctures in interstices. Sculpture of T6 in female: densely longitudinally striate, with fine punctures in interstices. Length of T6 in female: distinctly longer than wide. Apex of T6 in female: round. Sculpture of S2: longitudinally striate rugose. Sculpture of T1 in male: densely longitudinally striate, punctate rugulose in interstices. Male T8 apical spine: present.

**Figures 1–6.**
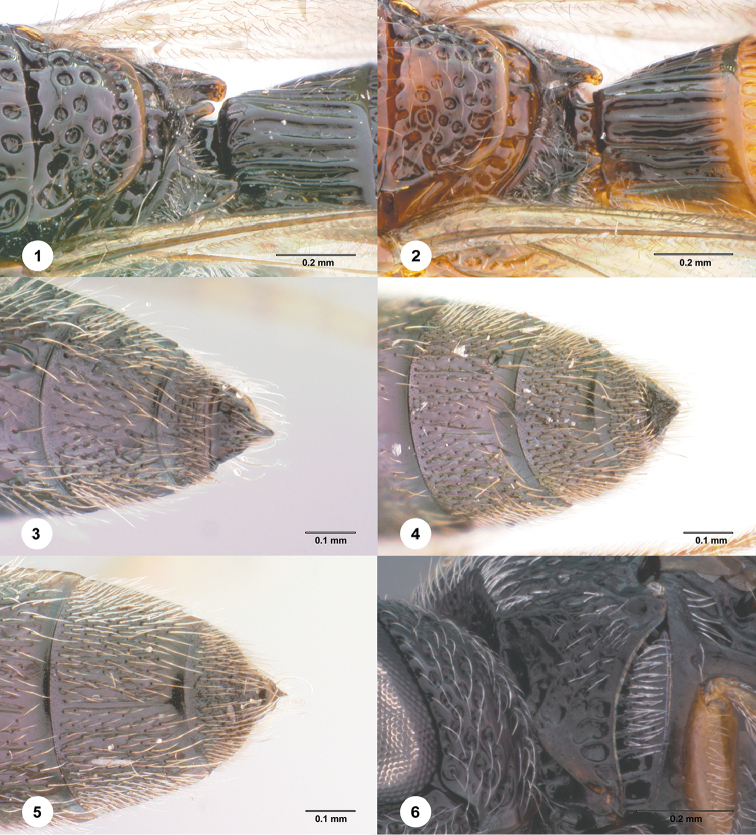
**1–2** *Habroteleia mutabilis* sp. n. **1** Paratype (FBA 143219), Propodeum, dorsolateral view **2** Holotype (FBA 070892), Propodeum, dorsolateral view **3** *Habroteleia salebra* sp. n., male, paratype (OSUC 688063), Apex of metasoma, dorsal view **4** *Habroteleia spinosa* sp. n., male, paratype (OSUC 232878), Apex of metasoma, dorsal view **5** *Habroteleia spinosa* sp. n., female, holotype (OSUC 232889), Apex of metasoma, dorsal view **6** *Habroteleia flavipes*, male (OSUC 58007), Pronotum, lateral view.

**Figures 7–10.**
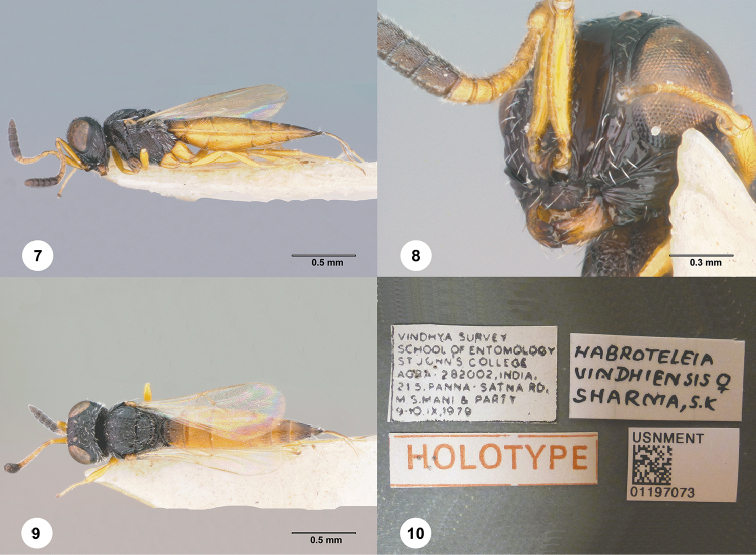
*Baryconus vindhiensis*, female, holotype (USNMENT01197073). **7** Lateral habitus **8** Head, lateral view **9** Dorsal habitus **10** Labels.

#### Diagnosis.

This species is most similar to *H. persimilis* but can be distinguished by its short median propodeal projection and longitudinally striate T6 in female.

#### Link to distribution map.

[http://hol.osu.edu/map-large.html?id=4535]

**Figures 11–16.**
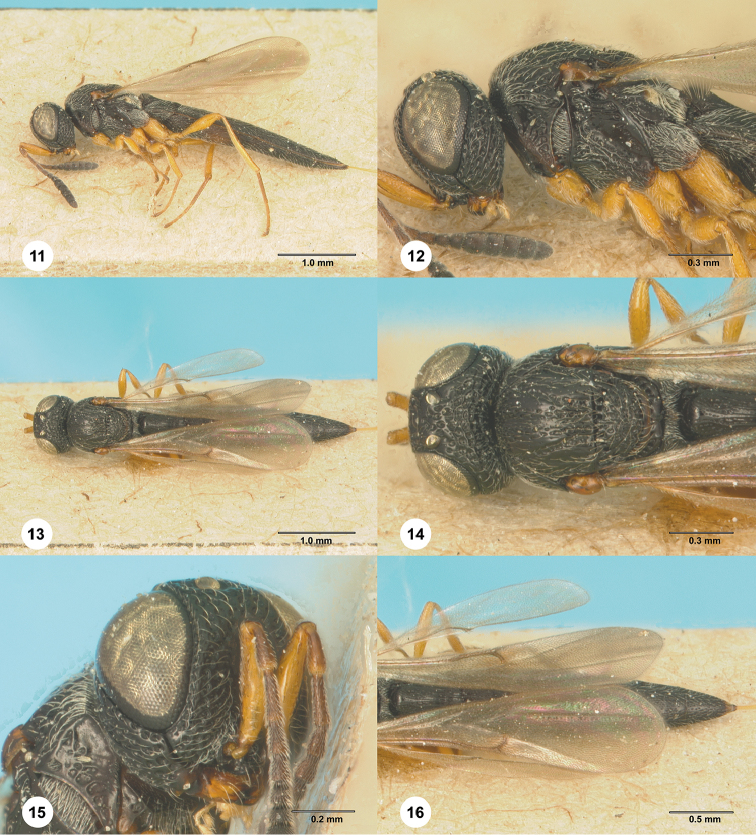
*Habroteleia flavipes*, female, holotype (MCSN 0001). **11** Lateral habitus **12** Head and mesosoma, lateral view **13** Dorsal habitus **14** Head and mesosoma, dorsal view **15** Head, lateral view **16** Metasoma and wings, dorsal view.

**Figures 17–22.**
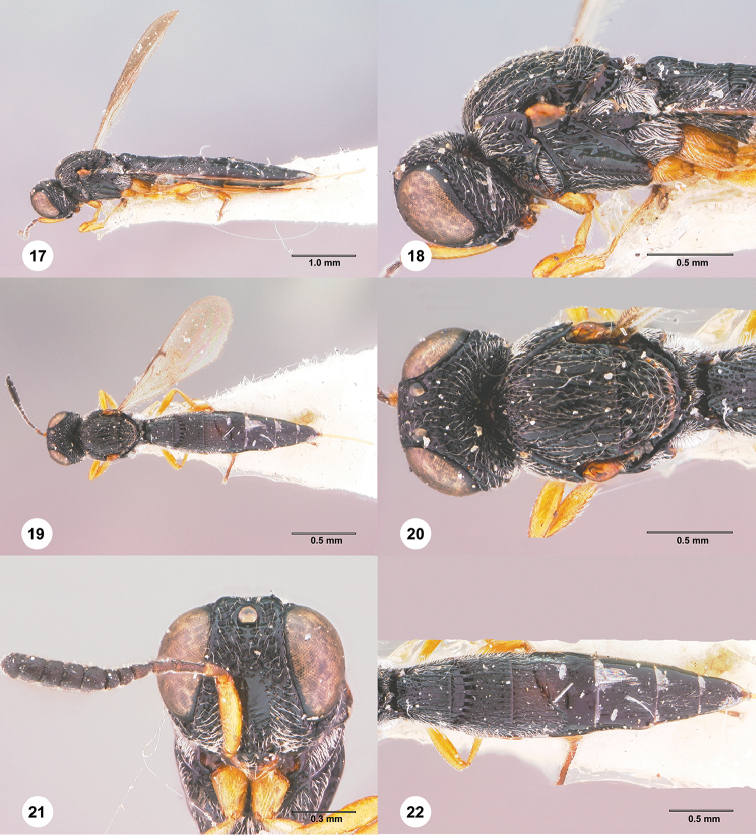
*Habroteleia bharatensis*, female, holotype (USNMENT01197132). **17** Lateral habitus **18** Head and mesosoma, lateral view **19** Dorsal habitus **20** Head and mesosoma, dorsal view **21** Head, anterior view **22** Metasoma, dorsal view.

#### Material examined.

Holotype of *Habroteleia flavipes* Kieffer, female: **INDONESIA**: Sumatera Utara Prov., Sumatra Isl., Pangherang Pisang, X.1890 – III.1891, E. Modigliani, MCSN 0001 (deposited in MCSN). Holotype of *Habroteleia bharatensis* Saraswat, female: **INDIA**: West Bengal St., 16.4, Poro North, 6.IV–24.IV.1976, M. S. Mani, USNMENT01197132 (deposited in USNM). Syntype of *Chrestoteleia bakeri* Kieffer, female: **PHILIPPINES**: Laguna Prov., Los Baños, no date, Baker, ANIC DB 32-020728 (deposited in ANIC). Syntype of *Chrestoteleia Bakeri* Kieffer, female: **PHILIPPINES**: Laguna Prov., Los Baños, no date, Baker, MNHN 0013 (deposited in MNHN). Holotype of *Habroteleia browni* Crawford, female: **PHILIPPINES**: Metropolitan Manila Reg., Manila, no date, R. Brown, USNM Type No. 12894 (deposited in USNM). Holotype of *Triteleia kotturensis* Sharma, female: **INDIA**: Kerala St., 24.8, Kottur, 4.X.1980, M. S. Mani et al., USNMENT01197074 (deposited in USNM). *Other material*: (137 females, 79 males, 1 unknown) **BANGLADESH**: 2 females, OSUC 688056–688057 (CNCI). **BRUNEI**: 1 female, OSUC 232932 (BPBM). **CAMBODIA**: 1 female, OSUC 232935 (BPBM). **CHINA**: 23 females, 7 males, OSUC 232920 (BPBM); SCAU 2010100389, 2010100402, 2010100419, 2010100431, 2010100437, 2010100445–2010100446, 2010100459, 2010100464, 2010100495, 2010100497, 2010100499, 2010100502, 2010100504–2010100505, 2010100508–2010100512, 2010100514, 2010100517–2010100518, 2010100521–2010100522, 2010100524–2010100526, 2010100552 (SCAU). **INDIA**: 1 male, OSUC 688053 (CNCI). **INDONESIA**: 58 females, 23 males, OSUC 232906 (BPBM); OSUC 687960–688009, 688014–688041 (CNCI); OSUC 58007–58008 (OSUC). **LAOS**: 2 females, 3 males, OSUC 687955–687959 (CNCI). **MALAYSIA**: 23 females, 26 males, OSUC 232907–232914, 232916–232919, 232923, 232931, 232933-232934, 232937, 246583 (BPBM); OSUC 687944–687954, 688058-688059 (CNCI); OSUC 491881–491896, 536427 (OSUC); OSUC 179084 (UCDC). **PHILIPPINES**: 1 female, 3 males, OSUC 232925–232928 (BPBM). **SOUTH KOREA**: 7 females, 2 males, 1 unknown, OSUC 687939 (CNCI); USNMENT01335741, 01335743–01335745, 01335747–01335749 (FSCA); USNMENT01335740, 01335742 (OSUC). **SRI LANKA**: 1 male, OSUC 688055 (CNCI). **THAILAND**: 16 females, 13 males, OSUC 232921–232922, 232924 (BPBM); OSUC 688042, 688049–688051 (CNCI); OSUC 321998–322002, 370249, 374199–374201, 381766–381770, 688080–688087 (OSUC). **VIETNAM**: 3 females, OSUC 232915 (BPBM); OSUC 688052 (CNCI); OSUC 284756 (OSUC).

#### Comments.

The metasomal color in *H. flavipes* varies from entirely dark brown to having T3–T4 and S2–S5 mostly yellow, apparently without any correlation with geography. In males, the length of the spine at the apex of T8 varies from short to long, but it is always present.

**Figures 23–28.**
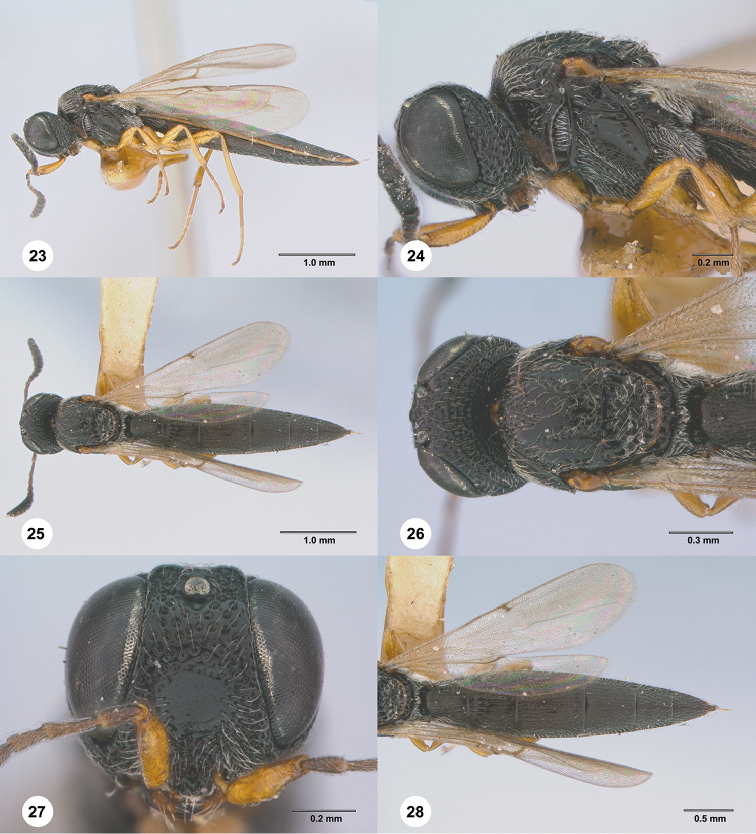
*Habroteleia browni*, female, holotype (USNM Type No. 12894). **23** Lateral habitus **24** Head and mesosoma, lateral view **25** Dorsal habitus **26** Head and mesosoma, dorsal view **27** Head, anterior view **28** Metasoma and wings, dorsal view.

**Figures 29–34.**
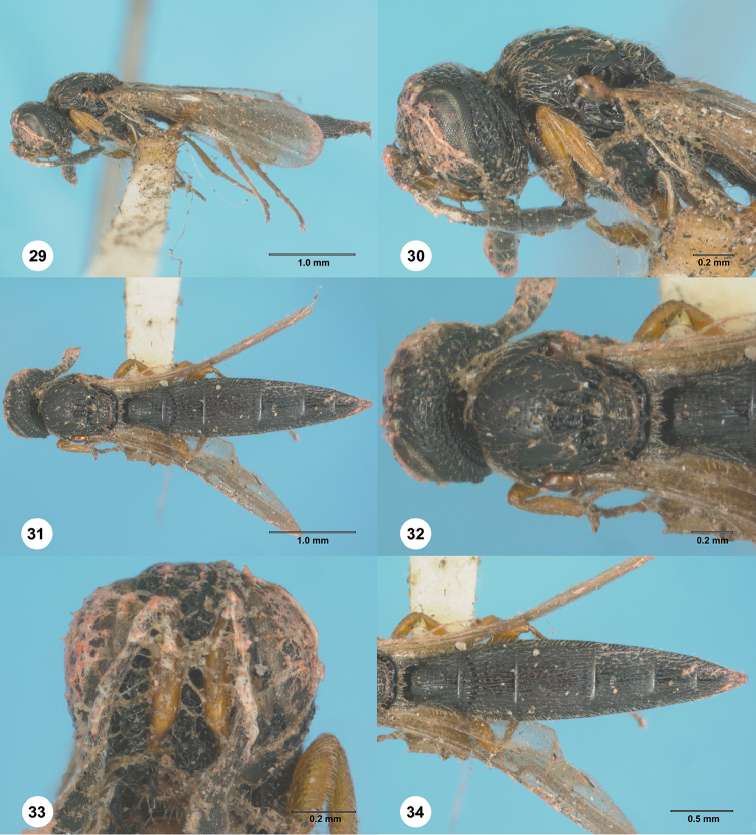
*Chrestoteleia bakeri*, female, holotype (MNHN 0013). **29** Lateral habitus **30** Head and mesosoma, lateral view **31** Dorsal habitus **32** Head and mesosoma, dorsal view **33** Head, anterior view **34** Metasoma and wings, dorsal view.

**Figures 35–40.**
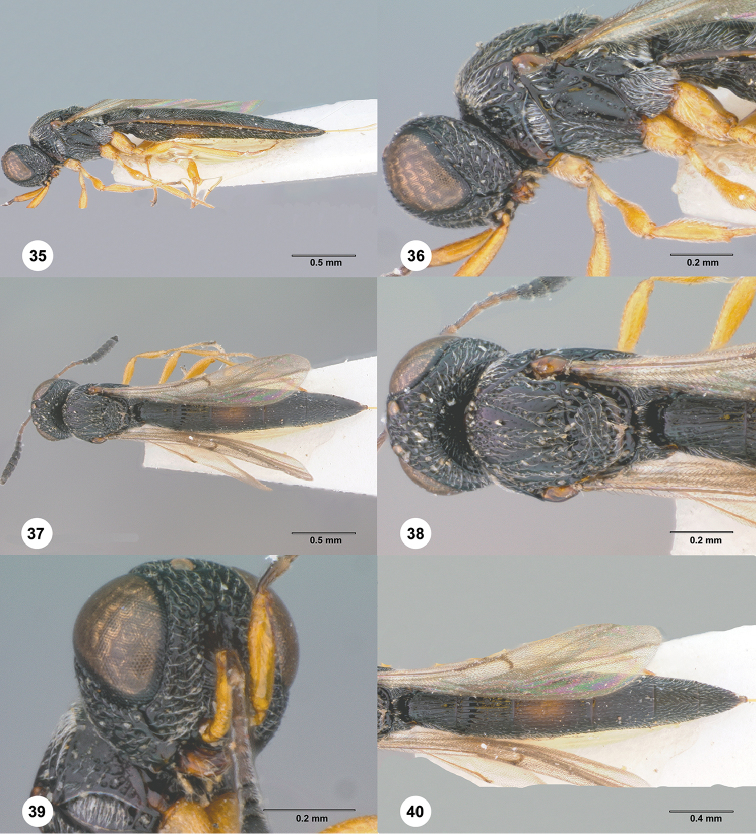
*Habroteleia kotturensis*, female, holotype (USNMENT01197074). **35** Lateral habitus **36** Head and mesosoma, lateral view **37** Dorsal habitus **38** Head and mesosoma, dorsal view **39** Head, lateral view **40** Metasoma and wings, dorsal view.

### Habroteleia impressa

Taxon classificationAnimaliaHymenopteraPlatygastridae

(Kieffer)

http://zoobank.org/5A7AAB83-B2A4-401F-A137-D96D29D7648E

http://bioguid.osu.edu/xbiod_concepts/4536

Crestoteleia impressa Kieffer, 1916: 180, 181 (original description, keyed, spelling error).
Chrestoteleia impressa Kieffer: Kieffer 1926: 443 (description, keyed); Baltazar 1966: 182 (cataloged, distribution).

Habroteleia impressa (Kieffer): Baltazar 1966: 177 (cataloged, generic transfer, distribution); Johnson 1992: 399 (cataloged, type information).

#### Comments.

We were unable to locate the type specimens of this species, and its status and identity are unclear.

### Habroteleia mutabilis

Taxon classificationAnimaliaHymenopteraPlatygastridae

Chen & Talamas sp. n.

http://zoobank.org/5ADA1AD2-2B82-4314-A7A6-E65EBDBBE561

http://bioguid.osu.edu/xbiod_concepts/448460

Figures 1–2 , 41–46 , 47–52

#### Description.

Body length of female: 3.60–3.74 mm (n=20). Body length of male: 3.36–3.72 mm (n=20). Length of A3 in male: longer than A2. Punctation of frons above antennal scrobe: sparse. Sculpture of antennal scrobe: foveate. Central keel: absent. Sculpture of ventrolateral frons: denstly punctate. Occipital carina: complete. Sculpture of posterior vertex: punctate rugose. Sculpture of gena: punctate rugose ventrally, sparsely punctate dorsally. Sculpture of occiput: smooth.

Color of mesosoma: black; orange. Sculpture of dorsal pronotal area: punctate rugose. Sculpture of lateral pronotal area: smooth anteriorly, foveate posteriorly. Sculpture of netrion: coarsely striate. Setae of netrion: sparse throughout. Sculpture of notaulus: discretely punctate. Sculpture of mesoscutal midlobe: coarsely carinate with two rows of contiguous coarse punctures; largely smooth, with two rows of discrete coarse punctures. Sculpture of lateral lobe of mesoscutum: smooth. Sculpture of lateral propodeal area: foveate. Setation of mesoscutellum: sparse. Sculpture of mesoscutellum: sparsely punctate. Median propodeal projection: short; long. Mesopleural carina: distinct. Sculpture of mesepisternum anteroventral to mesopleural depression: smooth with a row of punctures along mesopleural carina. Sculpture of dorsal metapleural area: smooth. Sculpture of ventral metapleural area: smooth to foveate. Setation of ventral metapleural area: sparse. Color of legs: orange-yellow to dark brown; dark brown to black. Sculpture of hind coxa: smooth.

Color of metasoma: black; orange with dark brown to black patches. T1 horn in female: absent. Sculpture of posterior margin of T1 in female: sparsely longitudinally striate. Transverse sulcus on T2: absent. Sculpture of T2–T5: T2–T3 sparsely longitudinally striate throughout, T4–T5 smooth medially, longitudinally striate. Sculpture of T6 in female: smooth. Length of T6 in female: wider than long. Apex of T6 in female: round. Sculpture of S2: sparsely longitudinally striate. Sculpture of T1 in male: sparsely longitudinally striate, smooth in interstices. Male T8 apical spine: absent.

**Figures 41–46.**
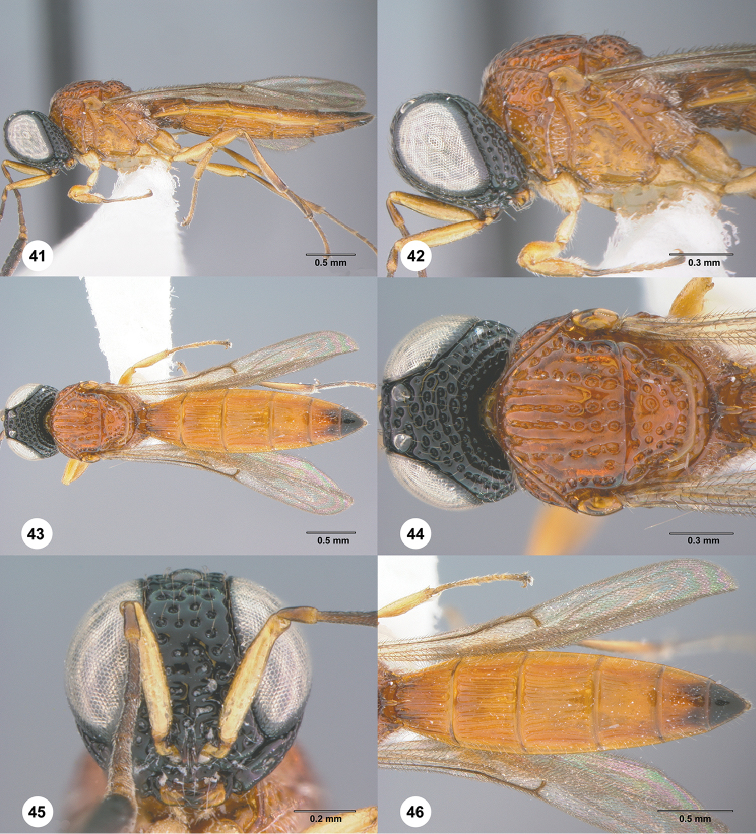
*Habroteleia mutabilis* sp. n., female, holotype (FBA 142603). **41** Lateral habitus **42** Head and mesosoma, lateral view **43** Dorsal habitus **44** Head and mesosoma, dorsal view **45** Head, anterior view **46** Metasoma and wings, dorsal view.

#### Etymology.

The epithet is inspired by the Latin word for changeable, in reference to the variations in body color, sculpture of mesoscutal midlobe, and the length of median propodeal projection, and is intended to be treated as an adjective.

#### Link to distribution map.

[http://hol.osu.edu/map-large.html?id=448460]

#### Material examined.

Holotype, female: **FIJI**: Northern Div., Bua Prov., Vanua Levu Isl., 6km NW Kilaka Village, MT5, 98m, 16.807°S, 178.991°E, Batiqere Range, 28.VI–21.VII.2004, Malaise trap, Schlinger & Tokota’a, FBA 142603 (deposited in BPBM). *Paratypes*: **FIJI**: 53 females, 27 males, FBA 070892, OSUC 232898, OSUC 232901, OSUC 232902, OSUC 232903, OSUC 232904, OSUC 232905 (BPBM); FBA 014394, 014404, 014409, 014413-014414, 019832, 025807, 025815, 029311, 029313, 029315, 029318-029320, 029323, 032077, 032086, 036322, 036328, 047849, 047855, 058998, 059005, 059026, 070887, 070893-070894, 082922, 084174-084175, 084181, 084183, 088442, 094483, 094485, 094487, 099217, 101111, 101129, 101568, 140945, 140956, 140961, 142806, 143124, 143130, 143134, 143209, 143217-143219, 144459, 151785, 151788, 164303, 166124, 166126, 166129, 166160, 166162-166163, 179833, 179838, 182136, 182139-182140, 182142, 186114, 188585, 188680, OSUC 688078, OSUC 688161, OSUC 688162 (CNCI). *Other material*: **FIJI**: 1 female, 1 male, OSUC 232900 (BPBM); FBA 084185 (CNCI).

#### Comments.

This species is well supported by many characters, although the color of mesosoma and metasoma, sculpture of mesoscutal midlobe, and the length of median propodeal projection are variable. The color of mesosoma and metasoma varies from orange to dark brown. The sculpture of the mesoscutal midlobe varies from largely smooth with two rows of discrete coarse punctures to coarsely carinate with two rows of contiguous coarse punctures. The length of the median propodeal projection varies from short to long. These variations are gradual among specimens. Therefore, we consider them as intraspecific rather than interspecific differences.

**Figures 47–52.**
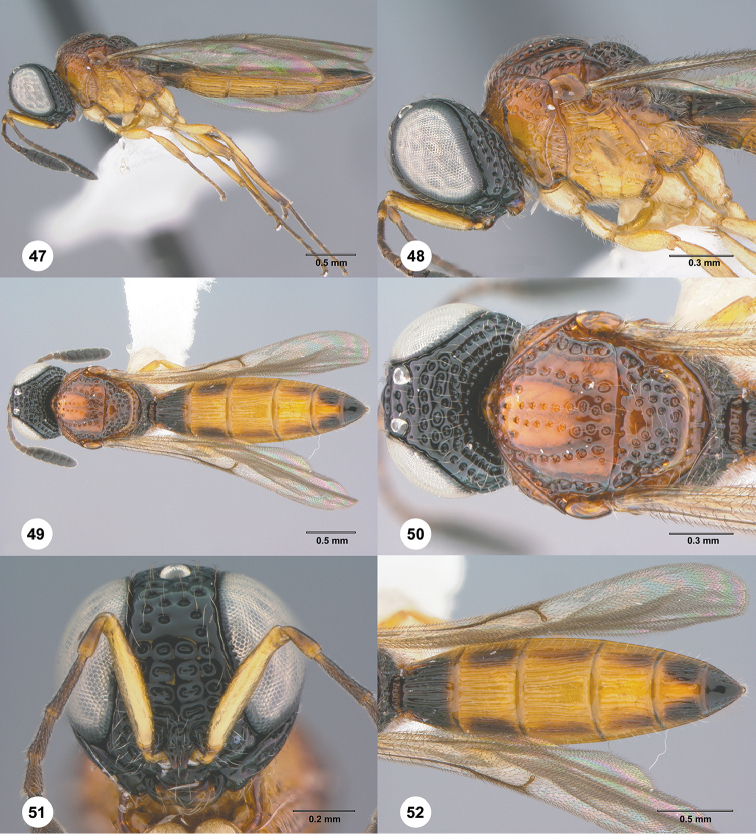
*Habroteleia mutabilis* sp. n., female, paratype (FBA 070892). **47** Lateral habitus **48** Head and mesosoma, lateral view **49** Dorsal habitus **50** Head and mesosoma, dorsal view **51** Head, anterior view **52** Metasoma and wings, dorsal view.

### Habroteleia persimilis

Taxon classificationAnimaliaHymenopteraPlatygastridae

(Kozlov & Kononova)

http://zoobank.org/F7A438F7-5207-4305-9467-11E23AA0923F

http://bioguid.osu.edu/xbiod_concepts/243852

Figures 53–58 , 59–64 , 89–90

Triteleia persimilis Kozlov & Kononova, 1985: 15, 17 (original description. Keyed); Kozlov and Kononova 1990: 174, 178 (description, keyed); Johnson 1992: 509 (cataloged, type information); Kononova 1995: 69 (keyed); Kononova and Petrov 2000: 28 (keyed).
Habroteleia persimilis (Kozlov & Kononova): Kononova and Kozlov 2008: 255 (description, generic transfer).
Triteleia dagavia Kozlov & Lê, 1995: 441, 445 (original description, keyed); Kozlov and Lê 1996: 9, 14 (described as new, keyed); Lê 2000: 76, 341 (description, keyed, type information), **syn. n.**
http://zoobank.org/451262B6-B23F-487F-A870-AAB91CB1E35A
http://bioguid.osu.edu/xbiod_concepts/28154
Habroteleia dagavia (Kozlov & Lê): Talamas and Pham 2017: 227 (type information, generic transfer).

#### Description.

Body length of female: 4.75–5.18 mm (n=20). Body length of male: 4.25–4.74 mm (n=20). Length of A3 in male: longer than A2. Punctation of frons above antennal scrobe: dense. Sculpture of antennal scrobe: punctate rugose to smooth. Central keel: present. Sculpture of ventrolateral frons: punctate rugose. Occipital carina: complete. Sculpture of posterior vertex: punctate rugose. Sculpture of gena: punctate rugose. Sculpture of occiput: densely finely punctate.

Color of mesosoma: black. Sculpture of dorsal pronotal area: punctate rugose. Sculpture of lateral pronotal area: smooth anteriorly, foveate posteriorly. Sculpture of netrion: coarsely striate. Setae of netrion: sparse throughout. Sculpture of notaulus: contiguously punctate. Sculpture of mesoscutal midlobe: largely densely punctate, with a medial furrow and smooth areas laterally. Sculpture of lateral lobe of mesoscutum: sparsely punctate. Sculpture of lateral propodeal area: rugose. Setation of mesoscutellum: dense. Sculpture of mesoscutellum: coarsely punctate rugose. Median propodeal projection: long. Mesopleural carina: distinct. Sculpture of mesepisternum anteroventral to mesopleural depression: punctate rugose. Sculpture of dorsal metapleural area: smooth. Sculpture of ventral metapleural area: rugose. Setation of ventral metapleural area: dense. Color of legs: orange-yellow. Sculpture of hind coxa: densely punctate.

Color of metasoma: black. T1 horn in female: present. Sculpture of posterior margin of T1 in female: largely longitudinally striate with horn punctate rugose. Transverse sulcus on T2: present. Sculpture of T2–T5: densely longitudinally striate, with fine punctures in interstices. Sculpture of T6 in female: densely punctate. Length of T6 in female: distinctly longer than wide. Apex of T6 in female: round. Sculpture of S2: densely longitudinally striate, punctate in interstices. Sculpture of T1 in male: densely longitudinally striate, punctate rugulose in interstices. Male T8 apical spine: absent.

**Figures 53–58.**
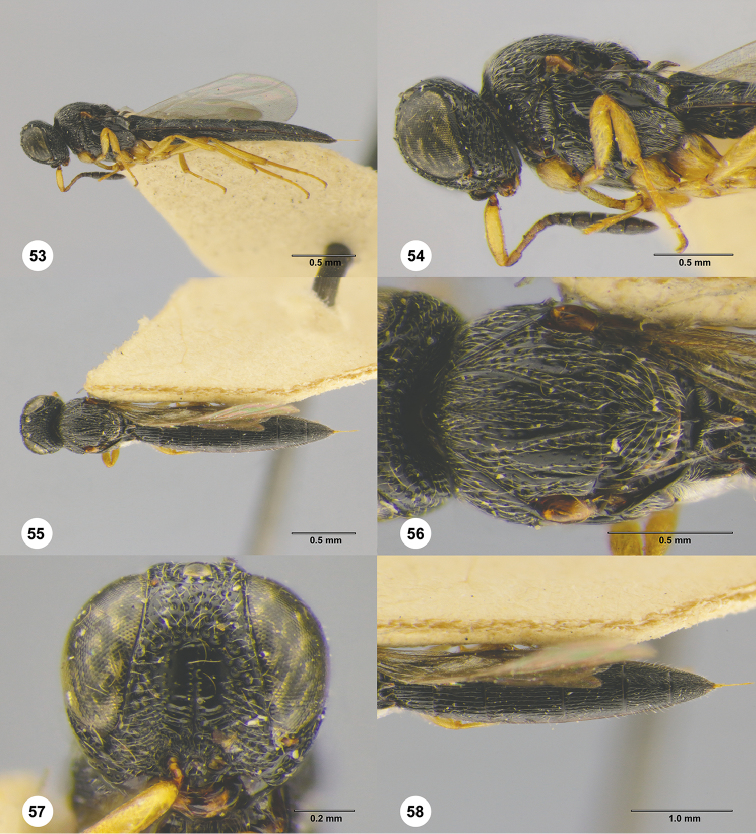
*Triteleia persimilis*, female, holotype (ZMAS 0139). **53** Lateral habitus **54** Head and mesosoma, lateral view **55** Dorsal habitus **56** Mesosoma, dorsal view **57** Head, anterior view **58** Metasoma, dorsal view.

#### Diagnosis.

This species is most similar to *H. flavipes* but can be distinguished by its long median propodeal projection and densely punctate T6 in female.

#### Link to distribution map.

[http://hol.osu.edu/map-large.html?id=243852]

#### Material examined.

Holotype, female, *T. persimilis*: **JAPAN**: Aichi Pref., Honshu Isl., Inuyama City, 6.X.1981, E. Sugonyaev, ZIN 0014 (deposited in ZIN). Holotype of *Triteleia dagavia* Kozlov & Lê, female: **VIETNAM**: Quang Nam Prov., Lang Stream, forest, Dak Pring, 31.X.1979, X. H. Lê, IEBR 0143 (deposited in IEBR). *Other material*: (48 females, 43 males) **CHINA**: 6 females, 10 males, SCAU 2010100315–2010100317, 2010100319–2010100320, 2010100322, 2010100330, 2010100335, 2010100337–2010100340, 2010100347, 2010100349, 2010100352–2010100353 (SCAU). **JAPAN**: 40 females, 29 males, OSUC 687863, 687865–687909, 687914–687936 (CNCI). **SOUTH KOREA**: 2 females, 4 males, OSUC 687937–687938, 687940–687943 (CNCI).

#### Comments.

*Habroteleia persimilis*, like *H. flavipes*, has a distribution that spans a large latitudinal range, extending from central Vietnam into the Palearctic region in Japan and South Korea.

**Figures 59–64.**
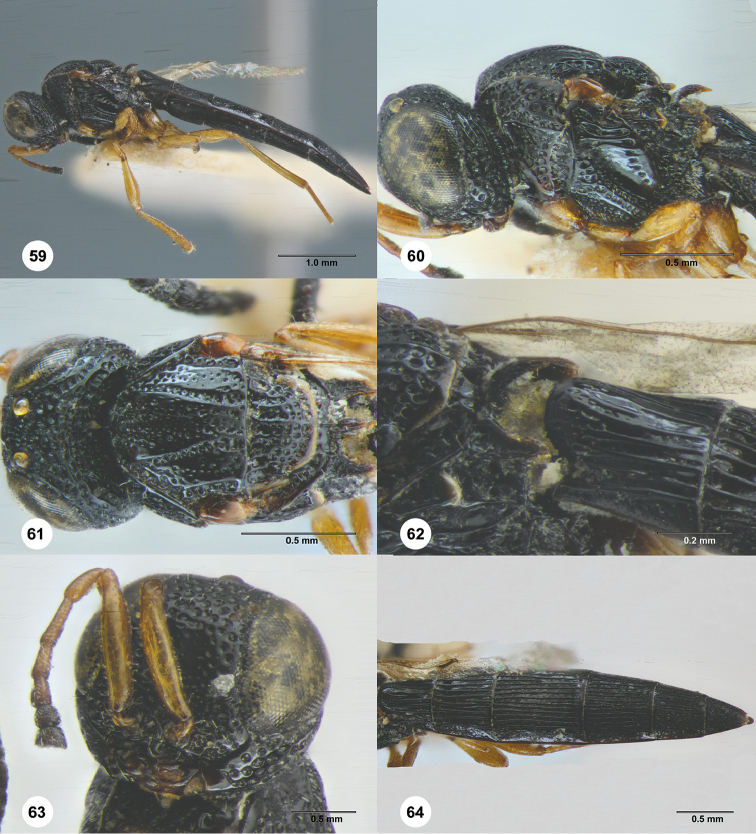
*Triteleia dagavia*, female, holotype (IEBR 0143). **59** Lateral habitus **60** Head and mesosoma, lateral view **61** Head and mesosoma, dorsal view **62** Propodeum, dorsolateral view **63** Head, lateral view **64** Metasoma, dorsal view.

### Habroteleia ruficoxa

Taxon classificationAnimaliaHymenopteraPlatygastridae

(Kieffer)

http://zoobank.org/C3EF6C6D-486C-47FC-B481-81EDF21FA806

http://bioguid.osu.edu/xbiod_concepts/4538

Figures 65–70

Phaedroteleia ruficoxa Kieffer, 1916: 182, 183 (original description. Keyed); Kieffer 1926: 418 (description, keyed); Kelner-Pillault 1958: 151 (type information); Baltazar 1966: 181 (cataloged, type information, distribution).
Habroteleia ruficoxa (Kieffer): Masner 1976: 26 (generic transfer); Johnson 1992: 399 (cataloged, type information).

#### Description.

Body length of male: 4.0 mm (n=1). Length of A3 in male: as long as A2.

Punctation of frons above antennal scrobe: sparse. Sculpture of antennal scrobe: foveate. Central keel: present. Sculpture of ventrolateral frons: punctate rugose. Occipital carina: complete. Sculpture of posterior vertex: punctate rugose. Sculpture of gena: punctate rugose. Sculpture of occiput: smooth.

Color of mesosoma: black. Sculpture of dorsal pronotal area: punctate rugose. Sculpture of lateral pronotal area: smooth anteriorly, foveate posteriorly. Sculpture of netrion: coarsely striate. Setae of netrion: dense throughout. Sculpture of notaulus: discretely punctate. Sculpture of mesoscutal midlobe: densely punctate. Sculpture of lateral lobe of mesoscutum: sparsely punctate. Sculpture of lateral propodeal area: rugose. Setation of mesoscutellum: sparse. Sculpture of mesoscutellum: sparsely punctate. Median propodeal projection: short. Mesopleural carina: distinct. Sculpture of mesepisternum anteroventral to mesopleural depression: largely smooth with sparse punctures. Sculpture of dorsal metapleural area: rugose. Sculpture of ventral metapleural area: rugose. Setation of ventral metapleural area: dense. Color of legs: orange-yellow. Sculpture of hind coxa: densely punctate.

Color of metasoma: black. Transverse sulcus on T2: present. Sculpture of T2–T5: sparsely longitudinally striate, smooth in interstices. Sculpture of T1 in male: sparsely longitudinally striate, smooth in interstices. Male T8 apical spine: absent.

#### Link to distribution map.

[http://hol.osu.edu/map-large.html?id=4538]

#### Material examined.

Holotype, male, *P. ruficoxa*: **PHILIPPINES**: Mindanao Isl., Butuan Chartered City, no date, Baker, MNHN_EY3427 (deposited in MNHN).

**Figures 65–70.**
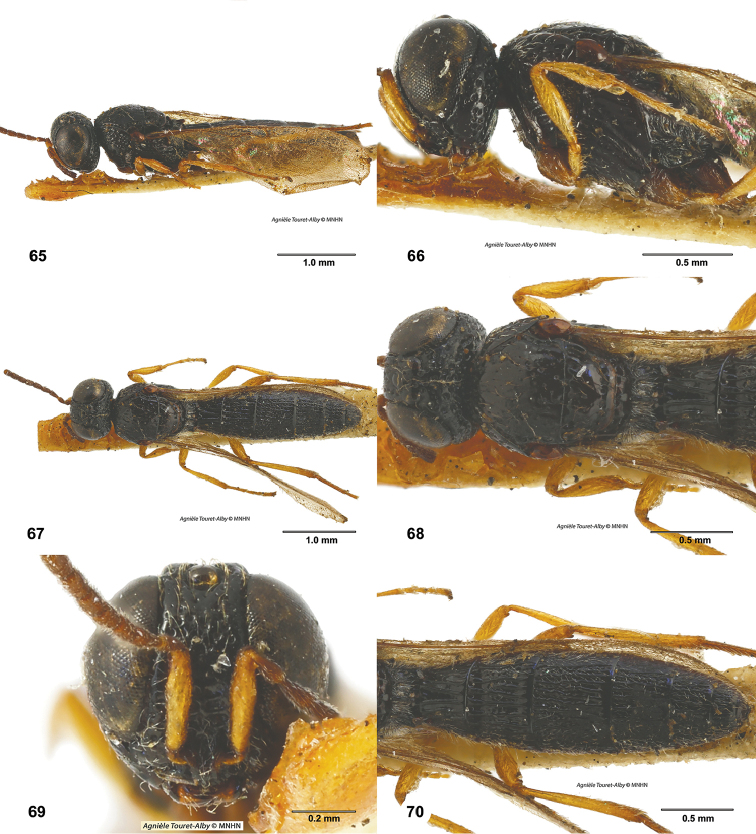
*Phaedroteleia ruficoxa*, male, holotype (MNHN_EY3427). **65** Lateral habitus **66** Head and mesosoma, lateral view **67** Dorsal habitus **68** Head and mesosoma, dorsal view **69** Head, anterior view **70** Metasoma, dorsal view.

#### Comments.

The holotype specimen of *Habroteleia ruficoxa* is in reasonably good condition in that the characters used for diagnosis at the species level are readily accessible. The challenge is that the species was described from a single male and in the course of this revision we did not encounter any additional specimens of *H. ruficoxa*. The absence of a spine on T8 in the male, the largely smooth surface of the mesoscutum and mesoscutellum, and the notauli weakly indicated by punctures place the holotype specimen well outside of our concept of *H. flavipes*, the only other species of *Habroteleia* known from the Philippines.

### Habroteleia salebra

Taxon classificationAnimaliaHymenopteraPlatygastridae

Chen & Talamas sp. n.

http://zoobank.org/F60BFB76-6AA1-4484-B2C7-CA6BE93CED9F

http://bioguid.osu.edu/xbiod_concepts/448456

Figures 3 , 71–76

#### Description.

Body length of female: 4.28–4.90 mm (n=20). Body length of male: 4.30–4.73mm (n=20). Length of A3 in male: longer than A2. Punctation of frons above antennal scrobe: sparse. Sculpture of antennal scrobe: smooth. Central keel: absent. Sculpture of ventrolateral frons: punctate rugose. Occipital carina: interrupted medially. Sculpture of posterior vertex: smooth with sparse punctures. Sculpture of gena: sparsely punctate. Sculpture of occiput: densely finely punctate.

Color of mesosoma: black. Sculpture of dorsal pronotal area: punctate rugose. Sculpture of lateral pronotal area: smooth anteriorly, foveate posteriorly. Sculpture of netrion: coarsely striate. Setae of netrion: sparse throughout. Sculpture of notaulus: discretely punctate. Sculpture of mesoscutal midlobe: densely and finely punctate along anterior margin, sparsely punctate along posterior margin, otherwise smooth. Sculpture of lateral lobe of mesoscutum: smooth. Sculpture of lateral propodeal area: rugose. Setation of mesoscutellum: sparse. Sculpture of mesoscutellum: coarsely punctate rugose. Median propodeal projection: short. Mesopleural carina: weakly developed. Sculpture of mesepisternum anteroventral to mesopleural depression: largely smooth with sparse punctures. Sculpture of dorsal metapleural area: smooth. Sculpture of ventral metapleural area: punctate rugose. Setation of ventral metapleural area: dense. Color of legs: dark brown to black. Sculpture of hind coxa: densely punctate.

Color of metasoma: black. T1 horn in female: present. Sculpture of posterior margin of T1 in female: densely longitudinally striate, punctate rugulose in interstices. Transverse sulcus on T2: present. Sculpture of T2–T5: densely longitudinally striate, with fine punctures in interstices. Sculpture of T6 in female: densely punctate. Length of T6 in female: distinctly longer than wide. Apex of T6 in female: round. Sculpture of S2: sparsely longitudinally striate medially, with fine punctures in interstices, irregularly finely punctate laterally. Sculpture of T1 in male: densely longitudinally striate, punctate rugulose in interstices. Male T8 apical spine: present.

**Figures 71–76.**
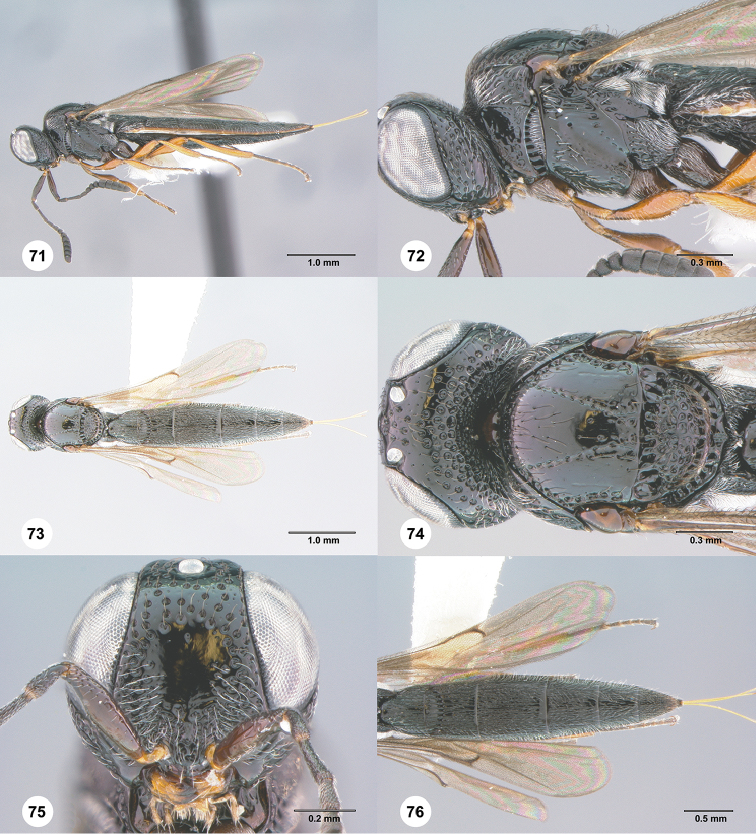
*Habroteleia salebra* sp. n., female, holotype (OSUC 688076). **71** Lateral habitus **72** Head and mesosoma, lateral view **73** Dorsal habitus **74** Head and mesosoma, dorsal view **75** Head, anterior view **76** Metasoma and wings, dorsal view.

#### Diagnosis.

This species is most similar to *H. spinosa* but can be distinguished by the round apex of T6 in females and the absence of a spine on the apex of T8 in males.

#### Etymology.

The epithet is inspired by the Latin word for a rough, uneven road, in reference to the glabrous netrion sulcus adjacent to the setose posterior portion of the netrion, and is intended to be treated as a noun in apposition.

#### Link to distribution map.

[http://hol.osu.edu/map-large.html?id=448456]

#### Material examined.

Holotype, female: **PAPUA NEW GUINEA**: Madang Prov., 100m, 04°16'S 144°58'E, Morox, 1.VIII–18.VIII.2006, yellow pan trap, V. Iwam, OSUC 688076 (deposited in CNCI). *Paratypes*: (21 females, 12 males) **INDONESIA**: 1 female, OSUC 232875 (BPBM). **PAPUA NEW GUINEA**: 20 females, 12 males, OSUC 232876–232877, 232879–232884, 232886, 232890–232892, 232894–232897 (BPBM); OSUC 688060–688063, 688065–688075, 688077 (CNCI).

### Habroteleia scapularis

Taxon classificationAnimaliaHymenopteraPlatygastridae

(Kieffer)

http://zoobank.org/6008D3A5-FE3A-4C26-8E5A-455A00D5DB9A

http://bioguid.osu.edu/xbiod_concepts/4539

Crestoteleia scapularis Kieffer, 1916: 180 (original description, keyed, spelling error).
Chrestoteleia scapularis Kieffer: Kieffer 1926: 443, 444 (description, keyed); Baltazar 1966: 182 (cataloged, distribution).
Habroteleia scapularis (Kieffer): Baltazar 1966: 177 (cataloged, generic transfer, distribution); Johnson 1992: 399 (cataloged, type information).

#### Comments.

We were not able to locate the type specimens of this species, and its status and identity are unclear.

### Habroteleia soa

Taxon classificationAnimaliaHymenopteraPlatygastridae

Chen & Talamas sp. n.

http://zoobank.org/DD68E31A-9B97-4226-832E-2549DD5F0E0A

http://bioguid.osu.edu/xbiod_concepts/448556

Figures 77–81

#### Description.

Body length of female: 3.72 mm (n=1). Length of A3 in male: longer than A2. Length of A3 in male: longer than A2. Punctation of frons above antennal scrobe: dense. Sculpture of antennal scrobe: smooth. Central keel: present. Sculpture of ventrolateral frons: punctate rugose. Occipital carina: complete. Sculpture of posterior vertex: punctate rugose. Sculpture of gena: punctate rugose ventrally, sparsely punctate dorsally. Sculpture of occiput: rugulose.

Color of mesosoma: black. Sculpture of dorsal pronotal area: punctate rugose. Sculpture of lateral pronotal area: smooth anteriorly, foveate posteriorly. Sculpture of netrion: coarsely striate. Setae of netrion: sparse throughout. Sculpture of notaulus: contiguously punctate. Sculpture of mesoscutal midlobe: punctate rugose on the anterior margin, otherwise largely smooth with two rows of discrete punctures. Sculpture of lateral lobe of mesoscutum: smooth. Sculpture of lateral propodeal area: rugose. Setation of mesoscutellum: sparse. Sculpture of mesoscutellum: coarsely punctate rugose. Median propodeal projection: short. Mesopleural carina: weakly developed. Sculpture of mesepisternum anteroventral to mesopleural depression: smooth with a row of punctures along mesopleural carina. Sculpture of dorsal metapleural area: smooth. Sculpture of ventral metapleural area: punctate rugose. Setation of ventral metapleural area: sparse. Color of legs: orange-yellow to dark brown. Sculpture of hind coxa: smooth.

Color of metasoma: black. T1 horn in female: absent. Sculpture of posterior margin of T1 in female: sparsely longitudinally striate. Transverse sulcus on T2: present. Sculpture of T2–T5: T2–T4 sparsely longitudinally striate, with fine punctures in interstices, T5 densely longitudinally striate punctate. Sculpture of T6 in female: densely punctate. Length of T6 in female: wider than long. Apex of T6 in female: round. Sculpture of S2: longitudinally striate rugose. Sculpture of T1 in male: sparsely longitudinally striate, smooth in interstices. Male T8 apical spine: present.

**Figures 77–81.**
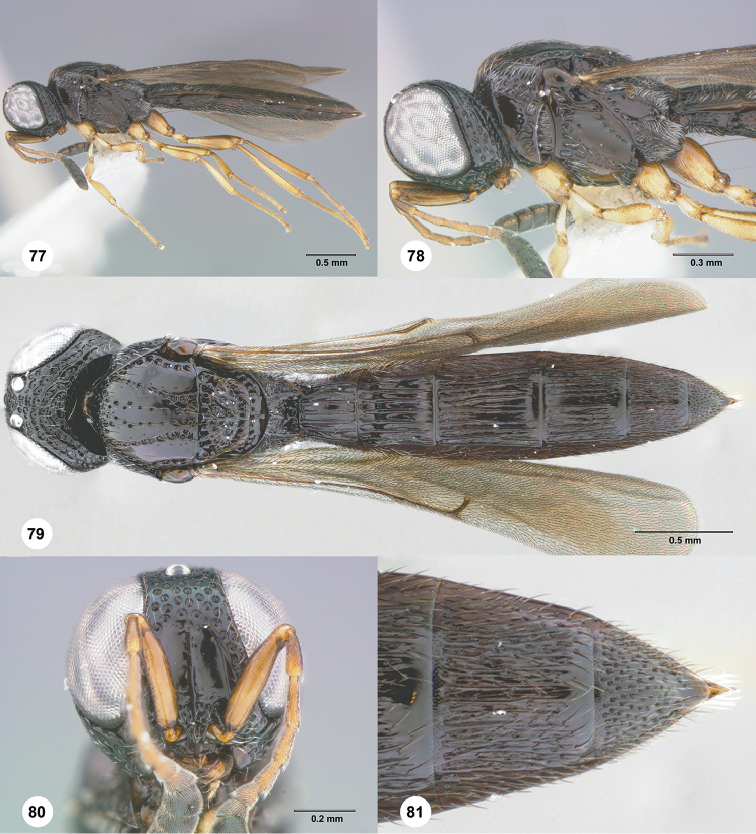
*Habroteleia soa* sp. n., female, holotype (CASENT 2136859). **77** Lateral habitus **78** Head and mesosoma, lateral view **79** Dorsal habitus **80** Head, anterior view **81** T5 and T6, dorsal view.

#### Etymology.

The Malagasy word “soa” means “beautiful” or “excellent”. We apply it to this species because we find it to be both of these. The name is treated as a noun in apposition.

#### Link to distribution map.

[http://hol.osu.edu/map-large.html?id=448556]

#### Material examined.

Holotype, female: **MADAGASCAR**: Antsiranana Auto. Prov., 5km W Manantenina, Camp Mantella, low altitude rainforest, MA-31-32, 490m, 14°26.29'S 49°46.44'E, Marojejy National Park, 14.X–22.X.2005, Malaise trap, M. Irwin & R. Harin’Hala, CASENT 2136859 (deposited in CAS). *Paratypes*: **MADAGASCAR**: 3 males, CASENT 2132434–2132435 (OSUC), 2135976 (CAS).

#### Comments.

*Habroteleia soa* is the most geographically disjunct member of the genus, separated from the other species by the Indian Ocean. Despite this separation, it is not morphologically unusual in comparison with the other species, suggesting either that there is a relatively recent division between *H. soa* and the other species, that the morphology of the genus evolves rather slowly, or that there has been insufficient sampling in the intervening areas (*e.g.*, east Africa, the moist southern part of the Arabian peninsula, India, and all other intervening regions).

### Habroteleia spinosa

Taxon classificationAnimaliaHymenopteraPlatygastridae

Chen & Johnson sp. n.

http://zoobank.org/9DD4E72F-B7E1-42CE-95BF-DDA22297830C

http://bioguid.osu.edu/xbiod_concepts/448458

Figures 4–5 , 82–87

#### Description.

Body length of female: 3.51–3.52 mm (n=2). Body length of male: 3.37–3.81 mm (n=6). Length of A3 in male: longer than A2. Punctation of frons above antennal scrobe: dense. Sculpture of antennal scrobe: punctate rugose. Central keel: present. Sculpture of ventrolateral frons: punctate rugose. Occipital carina: interrupted medially. Sculpture of posterior vertex: smooth with sparse punctures. Sculpture of gena: sparsely punctate. Sculpture of occiput: smooth.

Color of mesosoma: black. Sculpture of dorsal pronotal area: sparsely punctate. Sculpture of lateral pronotal area: largely smooth, with sparsely punctures medially. Sculpture of netrion: coarsely striate ventrally, rugulose dorsally. Setae of netrion: dense throughout. Sculpture of notaulus: discretely punctate. Sculpture of mesoscutal midlobe: densely finely punctate along anterior margin, otherwise smooth. Sculpture of lateral lobe of mesoscutum: smooth. Sculpture of lateral propodeal area: rugose. Setation of mesoscutellum: sparse. Sculpture of mesoscutellum: sparsely punctate. Median propodeal projection: short. Mesopleural carina: distinct. Sculpture of mesepisternum anteroventral to mesopleural depression: smooth. Sculpture of dorsal metapleural area: smooth. Sculpture of ventral metapleural area: rugose. Setation of ventral metapleural area: dense. Color of legs: dark brown to black. Sculpture of hind coxa: smooth.

Color of metasoma: black. T1 horn in female: absent. Sculpture of posterior margin of T1 in female: densely longitudinally striate, punctate rugulose in interstices. Transverse sulcus on T2: present. Sculpture of T2–T5: densely longitudinally striate, punctate rugulose in interstices. Sculpture of T6 in female: rugose. Length of T6 in female: wider than long. Apex of T6 in female: pointed. Sculpture of S2: sparsely longitudinally striate medially, with fine punctures in interstices, irregularly finely punctate laterally. Sculpture of T1 in male: sparsely longitudinally striate, smooth in interstices. Male T8 apical spine: absent.

**Figures 82–87.**
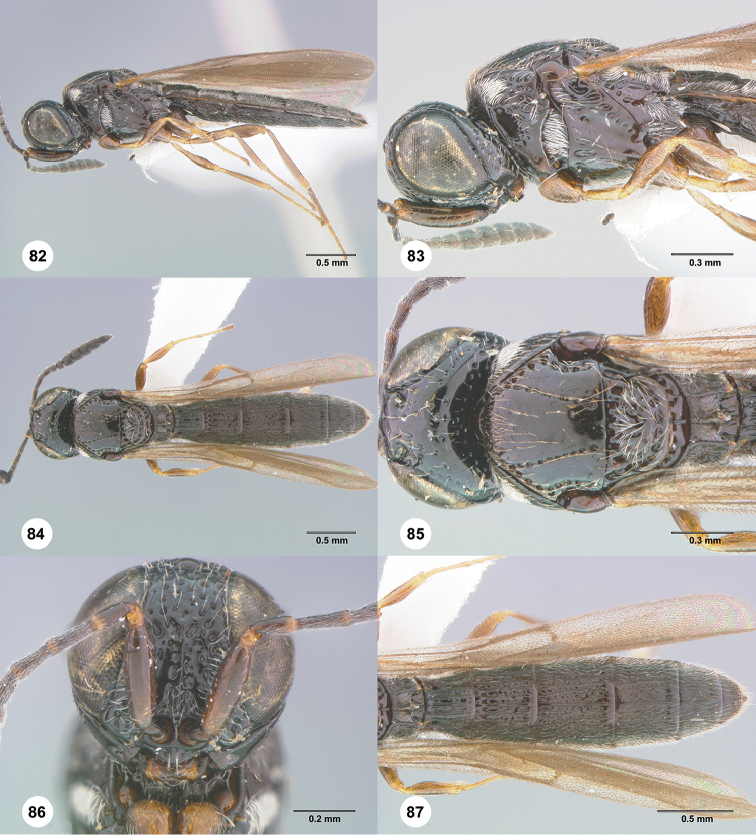
*Habroteleia spinosa* sp. n., female, holotype (OSUC 232889). **82** Lateral habitus **83** Head and mesosoma, lateral view **84** Dorsal habitus **85** Head and mesosoma, dorsal view **86** Head, anterior view **87** Metasoma and wings, dorsal view.

**Figures 88–90.**
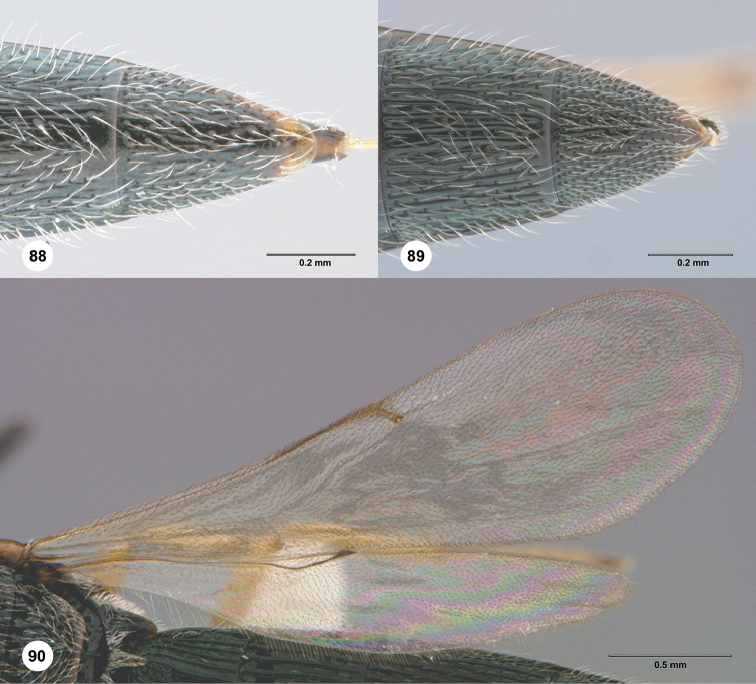
**88** *Habroteleia flavipes*, female (OSUC 688019), T5 and T6, dorsal view. **89–90** *Habroteleia persimilis*, female (OSUC 687934) **89** T5 and T6, dorsal view **90** Wings.

#### Diagnosis.

This species is most similar to *H. salebra* but can be distinguished by the pointed apex of T6 in females and the spine at the apex of T8 in males,

#### Etymology.

The specific epithet means spiny, referring to the pointed apex of T6 in females and should be treated as an adjective.

#### Link to distribution map.

[http://hol.osu.edu/map-large.html?id=448458]

#### Material examined.

Holotype, female: **INDONESIA**: Papua Prov., W New Guinea Isl., Central Mts., Archbold Lake, 760m, 26.XI–3.XII.1961, sweeping, L. W. Quate, OSUC 232889 (deposited in BPBM). *Paratypes*: (1 female, 6 males) **INDONESIA**: 6 males, OSUC 232878, 232887, 232893, 232929–232930, 234491 (BPBM). **PAPUA NEW GUINEA**: 1 female, OSUC 232888 (BPBM).

## Supplementary Material

XML Treatment for Habroteleia

XML Treatment for Baryconus vindhiensis

XML Treatment for Habroteleia flavipes

XML Treatment for Habroteleia impressa

XML Treatment for Habroteleia mutabilis

XML Treatment for Habroteleia persimilis

XML Treatment for Habroteleia ruficoxa

XML Treatment for Habroteleia salebra

XML Treatment for Habroteleia scapularis

XML Treatment for Habroteleia soa

XML Treatment for Habroteleia spinosa

## Appendix 1

**Table:**

	A1	http://purl.obolibrary.org/obo/HAO_0000908
	A2	http://purl.obolibrary.org/obo/HAO_0000706
	A3	http://purl.obolibrary.org/obo/HAO_0001148
	A7	http://purl.obolibrary.org/obo/HAO_0001885
	A12	http://purl.obolibrary.org/obo/HAO_0001884
	antenna	http://purl.obolibrary.org/obo/HAO_0000101
	antennomere	http://purl.obolibrary.org/obo/HAO_0000107
	area	http://purl.obolibrary.org/obo/HAO_0000146
	body	http://purl.obolibrary.org/obo/HAO_0000182
	carina	http://purl.obolibrary.org/obo/HAO_0000188
	central keel	http://purl.obolibrary.org/obo/HAO_0000109
cpa	cervical pronotal area	http://purl.obolibrary.org/obo/HAO_0000194
	clava	http://purl.obolibrary.org/obo/HAO_0000203
	clypeus	http://purl.obolibrary.org/obo/HAO_0000212
	compound eye	http://purl.obolibrary.org/obo/HAO_0000217
	coxa	http://purl.obolibrary.org/obo/HAO_0000228
	depression	http://purl.obolibrary.org/obo/HAO_0000241
dpa	dorsal pronotal area	http://purl.obolibrary.org/obo/HAO_0000267
	egg	http://purl.obolibrary.org/obo/HAO_0000286
	epomial carina	http://purl.obolibrary.org/obo/HAO_0000307
	eye	http://purl.obolibrary.org/obo/HAO_0000217
	femur	http://purl.obolibrary.org/obo/HAO_0000327
	fore wing	http://purl.obolibrary.org/obo/HAO_0000351
	frons	http://purl.obolibrary.org/obo/HAO_0001523
	gena	http://purl.obolibrary.org/obo/HAO_0000371
	head	http://purl.obolibrary.org/obo/HAO_0000397
dpa	hind coxa	http://purl.obolibrary.org/obo/HAO_0000587
	hind tibia	http://purl.obolibrary.org/obo/HAO_0000631
	hind wing	http://purl.obolibrary.org/obo/HAO_0000400
	inner orbit	http://purl.obolibrary.org/obo/HAO_0000419
	interantennal process	http://purl.obolibrary.org/obo/HAO_0000422
	lateral lobe of mesoscutum	http://purl.obolibrary.org/obo/HAO_0000466
	lateral ocellus	http://purl.obolibrary.org/obo/HAO_0000481
LOL	lateral ocellar line	http://purl.obolibrary.org/obo/HAO_0000480
lpa	lateral pronotal area	http://purl.obolibrary.org/obo/HAO_0000483
	malar sulcus	http://purl.obolibrary.org/obo/HAO_0000504
	mandible	http://purl.obolibrary.org/obo/HAO_0000506
	margin	http://purl.obolibrary.org/obo/HAO_0000510
	mesepisternum	http://purl.obolibrary.org/obo/HAO_0001872
med	mesopleural depression	http://purl.obolibrary.org/obo/HAO_0000326
	mesopleuron	http://purl.obolibrary.org/obo/HAO_0000566
	mesoscutellum	http://purl.obolibrary.org/obo/HAO_0000574
	mesoscutum	http://purl.obolibrary.org/obo/HAO_0001490
	mesosoma	http://purl.obolibrary.org/obo/HAO_0000576
	metapleuron	http://purl.obolibrary.org/obo/HAO_0000621
	metascutellum	http://purl.obolibrary.org/obo/HAO_0000625
	metasoma	http://purl.obolibrary.org/obo/HAO_0000626
	mesoscal midlobe	http://purl.obolibrary.org/obo/HAO_0000520
	netrion	http://purl.obolibrary.org/obo/HAO_0000644
	notauli (notaulus)	http://purl.obolibrary.org/obo/HAO_0000647
	occipital carina	http://purl.obolibrary.org/obo/HAO_0000653
	ocellus	http://purl.obolibrary.org/obo/HAO_0000661
ot	ocellar triangle	http://purl.obolibrary.org/obo/HAO_0000430
OOL	ocular ocellar line	http://purl.obolibrary.org/obo/HAO_0000662
	orbit	http://purl.obolibrary.org/obo/HAO_0000672
POL	posterior ocellar line	http://purl.obolibrary.org/obo/HAO_0000759
	process	http://purl.obolibrary.org/obo/HAO_0000822
	propodeum	http://purl.obolibrary.org/obo/HAO_0001248
	S1	http://purl.obolibrary.org/obo/HAO_0001997
	S2	http://purl.obolibrary.org/obo/HAO_0001829
	S3	http://purl.obolibrary.org/obo/HAO_0001831
	S4	http://purl.obolibrary.org/obo/HAO_0001832
	S5	http://purl.obolibrary.org/obo/HAO_0001833
	S6	http://purl.obolibrary.org/obo/HAO_0001834
	S7	http://purl.obolibrary.org/obo/HAO_0002185
	sculpture	http://purl.obolibrary.org/obo/HAO_0000913
	sternite	http://purl.obolibrary.org/obo/HAO_0001654
	sulcus	http://purl.obolibrary.org/obo/HAO_0000978
	T1	http://purl.obolibrary.org/obo/HAO_0000053
	T2	http://purl.obolibrary.org/obo/HAO_0000056
	T3	http://purl.obolibrary.org/obo/HAO_0000057
	T4	http://purl.obolibrary.org/obo/HAO_0000058
POL	T5	http://purl.obolibrary.org/obo/HAO_0000059
	T6	http://purl.obolibrary.org/obo/HAO_0000060
	T7	http://purl.obolibrary.org/obo/HAO_0000061
	tergite	http://purl.obolibrary.org/obo/HAO_0001783
	tibia	http://purl.obolibrary.org/obo/HAO_0001017
	tyloid	http://purl.obolibrary.org/obo/HAO_0001199
	vein	http://purl.obolibrary.org/obo/HAO_0001095
	vertex	http://purl.obolibrary.org/obo/HAO_0001077
	vertical epomial carina	http://purl.obolibrary.org/obo/HAO_0000307
	wing	http://purl.obolibrary.org/obo/HAO_0001089
